# Decreased Serum Levels of the Insulin Resistance-Related microRNA miR-320a in Patients with Polycystic Ovary Syndrome

**DOI:** 10.3390/cimb46040212

**Published:** 2024-04-15

**Authors:** Sarina Vogt, Diana Handke, Hermann M. Behre, Thomas Greither

**Affiliations:** Center for Reproductive Medicine and Andrology, Martin-Luther University Halle-Wittenberg, Ernst-Grube-Str. 40, 06120 Halle, Germany

**Keywords:** circulating microRNA, PCOS, insulin resistance, miR-320a

## Abstract

Polycystic ovary syndrome (PCOS) is often associated with metabolic abnormalities in the affected patients such as obesity or a dysregulated glucose metabolism/insulin resistance (IR). IR affects the serum levels of several circulating microRNAs; however, studies on the association between IR-related microRNAs and PCOS are scarce. Therefore, we quantified the serum levels of the IR-associated microRNAs miR-93, miR-148a, miR-216a, miR-224 and miR-320a via qPCR in a cohort of 358 infertility patients, of whom 136 were diagnosed with PCOS. In bivariate correlation analyses, the serum levels of miR-93 and miR-216a were inversely associated with dipeptidyl peptidase 4 serum concentrations, and the miR-320a serum levels were significantly downregulated in PCOS patients (*p* = 0.02, Mann–Whitney U test). Interestingly, in all patients who achieved pregnancy after Assisted Reproductive Technology (ART) cycles, the serum levels of the five IR-associated microRNAs were significantly elevated compared to those of non-pregnant patients. In cell culture experiments, we detected a significant upregulation of miR-320a expression following testosterone stimulation over 24 and 48 h in KGN and COV434 granulosa carcinoma cells. In conclusion, we demonstrated a significantly reduced serum level of the IR-associated miR-320a in our patient cohort. This result once again demonstrates the close relationship between metabolic disorders and the dysregulation of microRNA expression patterns in PCOS.

## 1. Introduction

The unfulfilled desire to have children is often caused by infertility, which is a common condition worldwide [1]. Infertility is defined as a condition characterized by the failure to achieve a clinical pregnancy after 12 months of regular, unprotected sexual intercourse or as a result of an impairment in a person’s ability to reproduce either individually or with a partner [2]. Worldwide, 8–12% of couples of reproductive age are affected [2]. A common cause of female infertility is advanced age; however, pathophysiological conditions such as endometriosis, tubal sterility due to infection or endocrinopathies such as PCOS are also common [3]. Polycystic ovary syndrome (PCOS) is a heteromorphic disorder with the diagnostic guiding criteria of oligomenorrhoea, hyperandrogenism and polycystic ovaries. According to the Rotterdam guidelines, at least two of these three criteria should be fulfilled for the diagnosis of PCOS [4]. PCOS has been shown to have a negative effect on female fertility and is therefore a cause of infertility [3,5,6]. Due to hormonal abnormalities, this endocrine disorder affects the ovarian cycle, resulting in oligomenorrhoea and ovulatory dysfunction [7]. In addition, metabolic abnormalities are a common feature of PCOS, which can manifest as obesity, metabolic syndrome and type 2 diabetes mellitus (T2DM). Even after the reproductive period, these conditions accompany the affected patient throughout her whole life and sometimes seriously affect the quality of life [8,9,10].

In particular, T2DM is a direct consequence of a prevalent insulin resistance (IR), which is an often-neglected feature of PCOS. In this regard, it should be mentioned that the serum concentration and activity of a therapeutic target of T2DM, namely, dipeptidyl peptidase 4 (DPP4), were previously shown by our group and others to be elevated in PCOS patients [11,12], while there are also reports showing that no differential DPP4 serum activity in PCOS exists [13,14].

In addition to the Rotterdam diagnostic criteria, insulin resistance is a key feature of PCOS patients and refers to the fact that peripheral tissue is less sensitive to insulin. This leads to an increase in insulin secretion and, hence, hyperinsulinism [15,16].

Since their discovery in the early 2000s, microRNAs have been shown to affect gene expression by regulating specific target mRNAs. As non-coding RNAs, microRNAs are approximately 22 nucleotides long and interact with their target mRNAs either by blocking translation or by degrading specific mRNAs [17]. Circulating microRNAs are secreted by exocytosis and can be transported by high-density lipoproteins or exosomes. MicroRNAs have been shown to be insensitive to heat or agitation (e.g., vigorous mixing) and may therefore be promising peripheral biomarkers [17].

Several circulating microRNAs in the serum of PCOS patients have been investigated and found to be associated with clinicopathological criteria of PCOS (reviewed in [18,19]). However, there is a lack of studies analyzing IR-associated circulating microRNAs as effectors of the PCOS.

Several IR-associated microRNAs have been described in the literature. One of the most prominent microRNAs, a reduction in miR-93, has been shown to increase adiposity and thus insulin resistance in mice via signaling pathways involving Tbx3 and sirt-7 [20]. Furthermore, the serum levels of miR-93 and miR-148, among others, differed significantly between individuals with pre-diabetes, those with T2DM and healthy controls [21]. More recently, the circulating microRNA miR-216a was described to be significantly reduced in obese women, and serum miR-216a levels were additionally associated with several metabolic parameters [22]. Reduced levels of miR-320 were also significantly associated with obesity in a cohort of Korean women [23]. In the granulosa cells of PCOS patients undergoing Assisted Reproductive Technology (ART) treatments, miR-320 was found to be downregulated, and the miR-320 expression was inversely correlated with insulin concentrations [24].

Therefore, the aim of this study was to evaluate the serum levels of the selected IR-associated microRNAs in patients with PCOS compared to control participants. Additionally, to gain mechanistic insights into associations between the PCOS phenotype characteristic hyperglycemia and hyperandrogenemia, we analyzed potential expression changes of the IR-associated microRNAs miR-93, miR-320a and miR-224 upon glucose or testosterone stimulation in granulosa carcinoma cells.

## 2. Materials and Methods

### 2.1. Patient Cohort and Preanalytics

Whole blood samples were collected from female patients treated for various causes of infertility at the Center for Reproductive Medicine and Andrology between 2010 and 2015. The study was conducted in accordance with the Declaration of Helsinki, and the study was approved by the Ethics Committee of the Martin Luther University Halle-Wittenberg (study numbers 218/14.04.10/2 and 2014-08). Written informed consent was obtained from all patients. Patient characteristics, including age, body mass index (BMI) and ovulation hormone concentrations, were recorded separately (see Table 1). Eight patients terminated the diagnostic process before the assignment of a definitive PCOS diagnosis. These patients were excluded from statistical comparisons regarding microRNA expression differences between PCOS and Non-PCOS patients. A total of 152 patients underwent ART cycles, of whom forty-five became pregnant (29.6%). Three of these patients had a miscarriage, while forty-two gave birth.

Whole blood was collected on day 8 of the menstrual cycle for women undergoing an ART cycle and during the first half of the menstrual cycle for women visiting the clinic for fertility diagnostics. After collection, the samples were immediately centrifuged at 900× *g* for 10 min to facilitate the separation into serum and cell fractions. Subsequently, the serum was then stored at −80 °C until RNA isolation.

### 2.2. RNA Isolation

RNA was isolated using Trizol (Life Technologies, Carlsbad, CA, USA). After the centrifugation of 400 µL serum, 700 µL Trizol was added to the supernatant and mixed. After five minutes of incubation at room temperature, 200 µL chloroform (AppliChem, Darmstadt, Germany) was added. The mixture was manually vortexed briefly and incubated for two minutes at room temperature. The samples were then centrifuged at 4 °C for 15 min. The supernatant was transferred and 2.5 µL DNase (Qiagen, Hilden, Germany) and 17.5 µL RDD-Buffer (Qiagen, Hilden, Germany) were added. After ten minutes of incubation at room temperature, 500 µL isopropanol (AppliChem, Darmstadt, Germany) was added and the samples were precipitated overnight at −20 °C. The precipitate was washed twice with ethanol (AppliChem, Darmstadt, Germany). In the first step, 900 µL of 96% ethanol was added, and in the second step, 900 µL of 70% ethanol was added. After drying, the precipitate was dissolved in 15 µL of RNase-free water (Fresenius, Bad Homburg, Germany).

### 2.3. cDNA Synthesis and qPCR

The miRCURY LNA RT Kit (Qiagen, Hilden, Germany) was used for cDNA synthesis. For one sample, the following amounts were added: 2 µL reaction Buffer, 4.5 µL H_2_O, 1 µL enzyme, 0.5 µL spike-in as an internal control and 2 µL of the previously prepared RNA-solution (20 ng/µL). The following steps were performed in a PCR cycler. The first step was 1 min at 42 °C; the second step was 5 min at 72 °C, followed by a cooling step. Quantitative real-time PCR (qPCR) was used to measure the serum levels of the selected microRNAs. Room temperature stable PCR reagents (Solis Biodyne, Tartu, Estonia) were used for the qPCR. For one sample, 2 µL of PCR reagents, 3 µL of water (Fresenius, Bad Homburg, Germany), 1 µL of a specific primer (Qiagen, Hilden, Germany) and 4 µL of the prepared cDNA sample were mixed. The following protocol was applied on the qPCR cycler (CFX96, BioRad, Hercules, CA, USA): 95 °C for twelve minutes, 95 °C for one minute, 60 °C for one minute, followed by a fluorescence reading, and steps 2–3 were repeated 45 times. A melting curve was then performed. Previous studies have suggested miR-16 as a reference gene for serum microRNA measurements [25,26]; therefore, miR-16 serum levels were quantified in our cohort. NormFinder analyses showed that miR-16 was stably expressed throughout the patient cohort, and therefore, miR-16 was selected as the reference gene.

### 2.4. Cell Culture

The experimental set-up consisted of two different granulosa carcinoma cell lines, the KGN cell line and the COV 434 cell line. For each flask, 200,000 cells per flask were cultured in medium DMEM (Gibco, Paisley, UK) supplemented with penicillin/streptomycin (PAA, Cölbe, Germany) and FCS (PAA, Cölbe, Germany). One flask per cell line was treated with one of the following fluids: cell medium only, 50 mM glucose (AppliChem, Darmstadt, Germany), 100 mM glucose (AppliChem, Darmstadt, Germany), DMSO (AppliChem, Darmstadt, Germany), 30 nM testosterone (Sigma Life Science, Steinheim, Germany) or 100 nM testosterone (Sigma, Steinheim, Germany). Cells were harvested after either 24 h or 48 h. All experiments were performed in triplicate.

### 2.5. Statistics

We used the program IBM SPSS statistics 28.0.0.0 (IBM Cooperation, New York, NY, USA) to analyze the retrieved data. The primary outcome was a diagnosis of PCOS according to the Rotterdam criteria. Secondary endpoints were each of the three PCOS diagnostic criteria, a clinical pregnancy after ART treatment and miscarriage/continued pregnancy. Bivariate correlation analyses were used to compare the serum levels of each. Comparisons between serum microRNA levels and clinical parameters were performed using the non-parametric Mann–Whitney U test, as the serum microRNA levels were not normally distributed. Differences in microRNA expression upon testosterone or glucose stimulation were analyzed by Student’s *t*-test, as these values were normally distributed. Results with a *p*-value below 0.05 were considered statistically significant.

## 3. Results

### 3.1. Serum Levels of IR-Associated microRNAs

**Figure 1:**
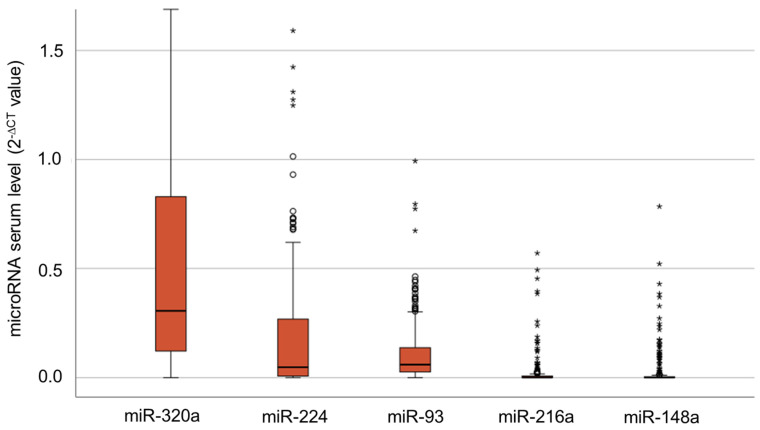
Serum levels of miR-320a, miR-224, miR-93, miR-216a and miR-148a in the total patient cohort. The *Y*-axis was cropped at 1.6 for better readability. Circles and stars represent mild and extreme outliers.

We analyzed the serum levels of each microRNA by qPCR. MicroRNA expression was calculated as 2^−∆CT^ [27]; therein, miR-16 expression was used as a reference gene. All microRNAs were detectable in our study cohort (see Figure 1), with higher levels of miR-93 (median: 0.06; 2^−∆CT^ values), miR-224 (median: 0.07) and miR-320a (median: 0.3). The serum levels of miR-93 ranged from 0.0 to 17.7, those of miR-224 ranged from 0.0 to 274.9 and those of miR-320a ranged from 0.0 to 230.7. MiR-148a (median: 0.0006) and miR-216a (median: 0.0004) were observed in a smaller proportion of patients and at much lower levels. MiR-148a showed a range between 0.0 and 0.9, and miR-216a showed a range from 0.0 to 0.8.

### 3.2. Correlation Analyses via Bivariate Correlation Analyses

We analyzed the correlation between different parameters using bivariate Spearman–Rho correlation analyses (see Table 2). The analyses showed significant associations between the serum levels of several microRNAs. MiR-93 serum levels were negatively correlated with the dipeptidyl peptidase 4 (DPP4) serum levels and positively correlated with miR-320a, miR-216a, miR-224 and miR-24 serum levels (each *p* ≤ 0.001). The serum levels of miR-320a correlated positively with the serum levels of either miR-222, miR-186, miR-93, miR-216a or miR-224 (each *p* ≤ 0.001). MiR-216a serum levels were negatively correlated with serum DPP4 activity (*p* = 0.002) and with the DPP4 serum concentration (*p* ≤ 0.001). Positive correlations were observed with estradiol, miR-93, miR-320a and miR-148a serum levels. The serum levels of miR-148a showed positive correlations with the serum levels of miR-216a (*p* ≤ 0.001). The serum levels of miR-224 showed a negative correlation with estradiol and a positive correlation with the serum levels of miR-93 and miR-224 (each *p* ≤ 0.001).

### 3.3. MiR-320a Serum Levels Are Associated with a PCOS Diagnosis

**Figure 2:**
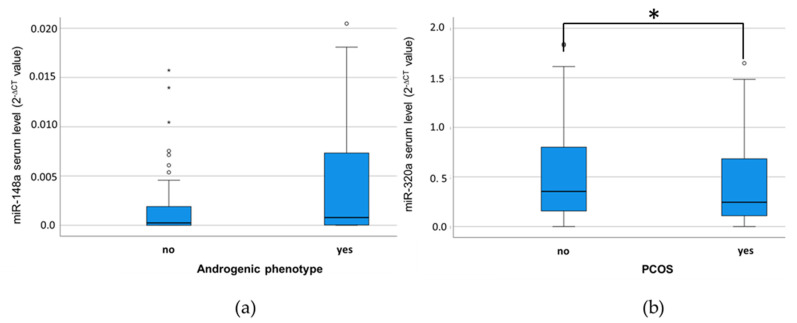
Trend towards an association between (**a**) miR-148a serum levels and the androgenic phenotype (*p* = 0.056). Significant association between (**b**) miR-320a serum levels and the occurrence of a PCOS (*p* = 0.021); Mann–Whitney U-Test; * *p* ≤ 0.05. Circles and stars represent mild and extreme outliers.

We analyzed the association between the serum levels of each microRNA and other parameters using the Mann–Whitney U test. Of the three diagnosis criteria for PCOS, no statistically significant results were observed for the criteria of dysmenorrhoea and polycystic ovaries on ultrasound. Elevated serum miR-148a levels showed a trend towards an association with the occurrence of an androgenic phenotype/hyperandrogenism, yet the significance level was not reached (*p* = 0.056; Mann–Whitney U-Test; see Figure 2a). However, when testing the distribution of the individual serum levels of the five selected microRNAs between PCOS and Non-PCOS patients, miR-320a serum levels were significantly lower in PCOS patients in comparison to Non-PCOS patients (*p* = 0.021; see Figure 2b).

**Figure 3:**
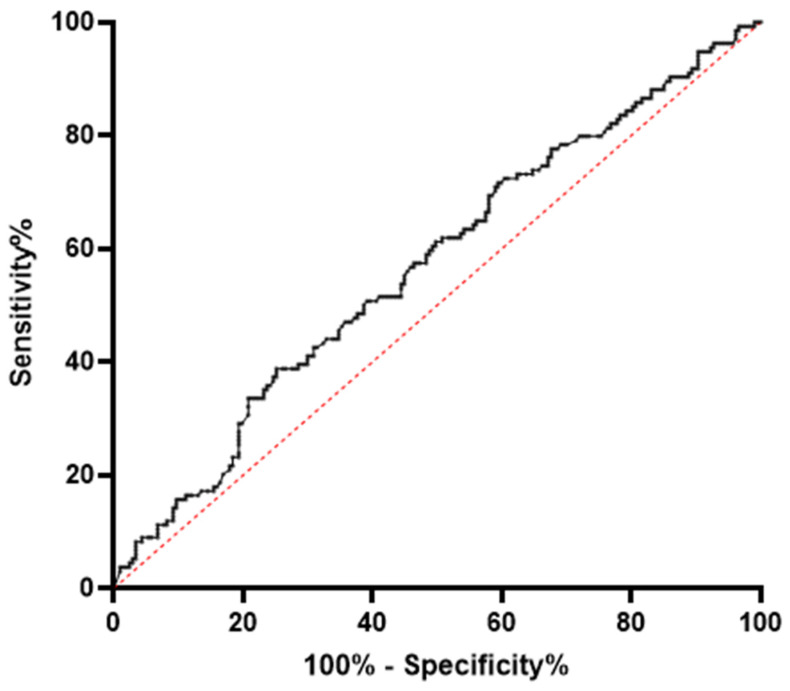
Receiver operating characteristics (ROC) curve analysis on the diagnostic potential of miR-320a serum levels in PCOS.

In the receiver operating characteristics, the miR-320a serum level showed significant power in distinguishing between the PCOS and the Non-PCOS group (asymptotic *p* = 0.03); however, the diagnostic potential was rather weak (AUC = 0.57; 95% CI = 0.51–0.63; see Figure 3).

When analyzing the association between the individual serum microRNA levels and the BMI, we did not observe significant associations between serum microRNAs and BMI ≥ 25 (overweight) or BMI ≥ 30 (obese) patients in Mann–Whitney U-tests.

Information on the usage of metformin was only available for a subset of patients. While 83 of the Non-PCOS patients did not use metformin, 20 Non-PCOS patients used metformin. Vice versa, 68 of the PCOS patients used metformin, while 17 did not. In non-parametric tests, in the whole subset, metformin usage was not significantly associated with altered serum microRNA serum levels (miR-93: *p* = 0.998; miR-320a: *p* = 0.181; miR-148a: *p* = 0.611; miR-216a: *p* = 0.985; miR-224: *p* = 0.213; Mann–Whitney U-test). Additionally, other than analyzing the PCOS or the Non-PCOS group, there was no association between metformin usage and the individual microRNA serum levels.

**Figure 4:**
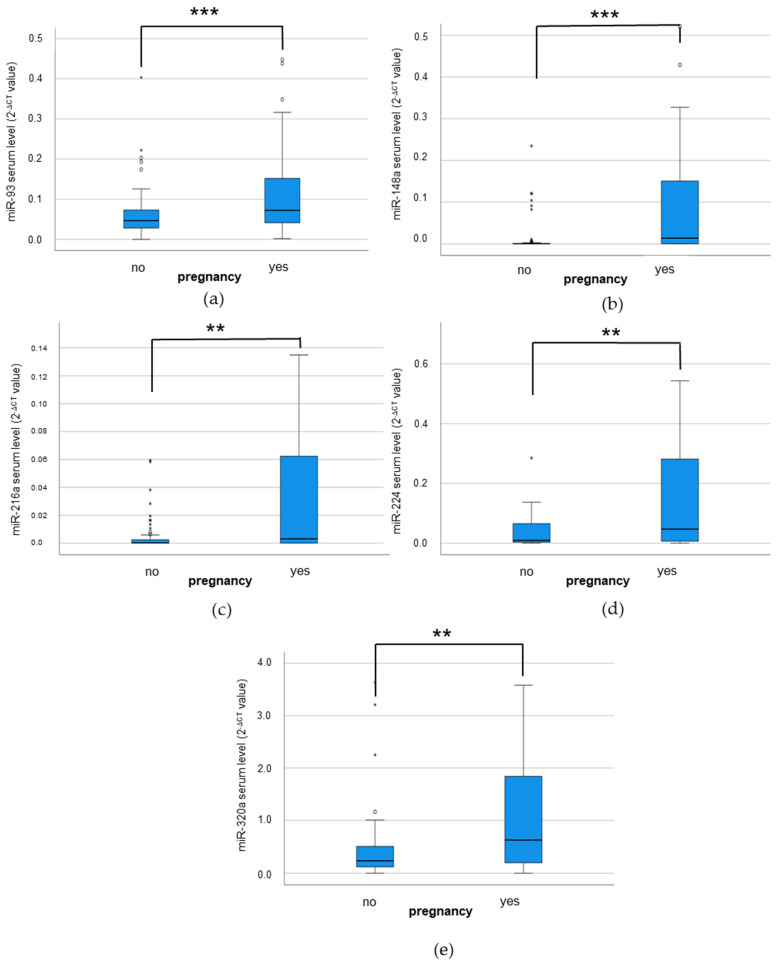
Association between microRNA serum levels and pregnancy. (**a**) miR-93 (*p* ≤ 0.001); (**b**) miR-148a (*p* = 0.001); (**c**) miR-216a (*p* = 0.002); (**d**) miR-224 (*p* = 0.015); (**e**) miR-320a (*p* = 0.016); Mann–Whitney U-Test; ** *p* ≤ 0.01; *** *p* ≤ 0.001. Circles and stars represent mild and extreme outliers.

An increased serum level of each microRNA examined was significantly associated with the occurrence of a pregnancy after ART (miR-93: *p* ≤ 0.001; miR-148a: *p* = 0.001; miR-216a: *p* = 0.002; miR-224: *p* = 0.015; miR-320a: *p* = 0.016; Mann–Whitney U-Test; see Figure 4).

### 3.4. Analysis of IR-Associated microRNA Expression under Hyperglycemic/Hyperandrogenemic Conditions

To detect the potential mechanistic interactions between altered microRNA expressions induced by PCOS-associated hyperglycemia or hyperandrogenemia, we aimed-for in vitro analyses in an ovarian cell model that is equally affected by increased androgen or glucose concentrations. Therefore, we cultivated the granulosa carcinoma cell lines COV434 and KGN with different concentrations of testosterone or glucose for a duration of 24 or 48 h. Subsequently, we analyzed the expressions of miR-93, miR-320a and miR-224, which, in our previous analyses, were elevated in the serum of our PCOS cohort in the treated cell lines in comparison to untreated controls via qPCR.

**Figure 5:**
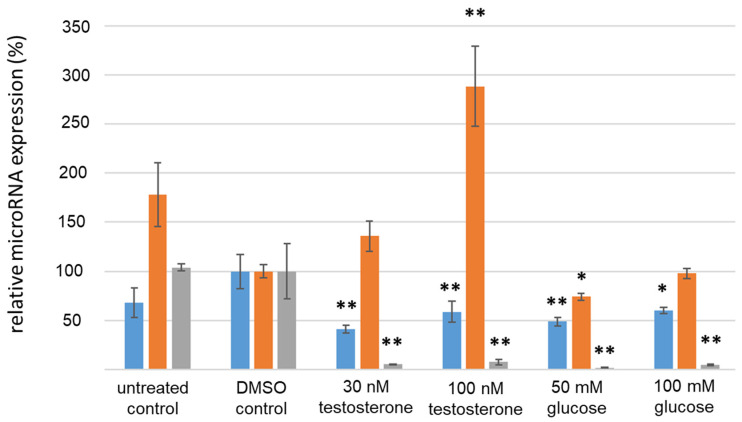
MicroRNA expression in KGN cells after 24 h treatment. Blue bars indicate miR-93 expression, orange bars indicate miR-320a expression and grey bars indicate miR-224 expression. * *p* ≤ 0.05; ** *p* ≤ 0.01; Student’s *t*-test.

The cell culture experiments in KGN granulosa carcinoma cells showed alterations in microRNA expression after 24 h when stimulated with glucose or testosterone compared to the DMSO treatment (see Figure 5). MiR-93 expression was consistently downregulated by 41% to 60% when treated with either glucose or testosterone (*p* ≤ 0.01 each, Student’s *t*-test). MiR-320a expression was significantly upregulated by approximately 180% when treated with 100 nM testosterone. An upregulation by 43% after stimulation with 30 nM testosterone was also observed; however, this effect was not significant (*p* > 0.05; *t*-Test). MiR-224 was significantly reduced by 92% to 98% when stimulated with either glucose or testosterone (*p* ≤ 0.01, Student’s *t*-test).

**Figure 6:**
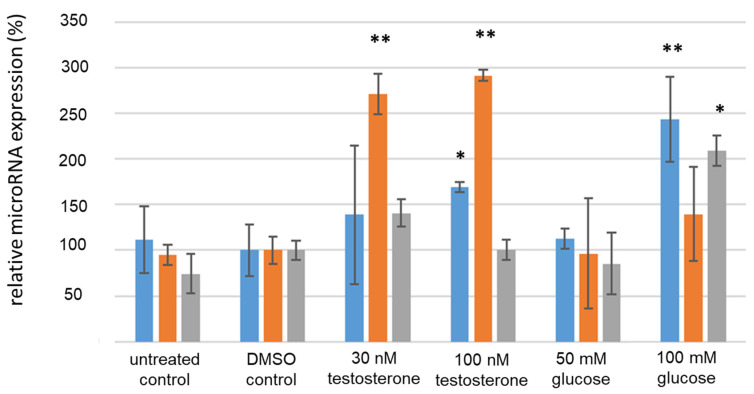
MicroRNA expression in KGN cells after 48 h treatment. Blue bars indicate miR-93 expression, orange bars indicate miR-320a expression and grey bars indicate miR-224 expression. * *p* ≤ 0.05; ** *p* ≤ 0.01; Student’s *t*-test.

After 48 h, the serum levels of the treated KGN cells were analyzed by qPCR. When treated with glucose and testosterone, the cells showed changes in microRNA expression in comparison to the DMSO control (see Figure 6). MiR-93 was significantly upregulated by approximately 55% when stimulated with 100 nM testosterone and by 145% when stimulated with 100 mM glucose (*p* ≤ 0.05, Student’s *t*-test). MiR-320a remained to be significantly upregulated by 180% to 200% when stimulated with testosterone (*p* ≤ 0.01, Student’s *t*-test). MiR-224 was significantly upregulated by 109% when stimulated with 100 mM glucose (*p* ≤ 0.05, Student’s *t*-test). In COV434 cells, the relative expression of the IR-associated microRNA examined under hyperglycemic or hyperandrogenemic conditions, especially regarding the significant upregulation of miR-320a upon testosterone stimulation over 24–48 h, was similar to that in KGN cells.

## 4. Discussion

In this study, we analyzed the serum expression of the IR-associated microRNAs miR-93, miR-148a, miR-216a, miR-224 and miR-320a in a cohort of 136 PCOS patients in comparison to 214 Non-PCOS control participants by qPCR. Additionally, we tested the effects of hyperglycemic and hyperandrogenemic environments on the expression of the three most abundant microRNAs, miR-93, miR-224 and miR-320a, in vitro.

In the whole study cohort, miR-216a and miR-148a showed lower serum levels, whereas miR-93, miR-224 and miR-320a exhibited higher serum levels. MiR-93 is described as consistently measurable circulating microRNA in several metabolism-related studies [28,29,30]. MiR-93 has been shown to be upregulated in women with PCOS and could therefore be a potential biomarker [31]. In addition, miR-93 may be associated with insulin resistance via regulation of the GLUT4 receptor [32]. An increase in miR-93 expression has been described following stimulation with insulin [33], although this finding is not supported consistently by the literature [31].

MiR-320a has also been reported as detectable circulating microRNA in different studies in regard to T2DM and insulin [34,35,36]. MiR-320a has been shown to be both downregulated and upregulated in the reproductive organs of women with PCOS. In cumulus granulosa cells, miR-320a was downregulated in a study of 21 patients [37]. In ovarian tissue from PCOS patients, miR-320a was found to be upregulated in one study, suggesting that this microRNA is a potential biomarker [38]. In addition, miR-320a was highly overexpressed in IR adipocytes, and the inhibition of miR-320a by AntagoMiRs increased the insulin sensitivity of the IR adipocytes [39]. We found that miR-224 had higher serum levels in our patients cohort, which is in line with previous studies [40,41]. Increased expression of this microRNA exhibited a correlation with lower oocyte quality and blastocyst development due to increased apoptosis, a possible indication of the correlation between polycystic ovaries in the PCOS and an altered miR-224 expression [42].

MiR-148a and miR-216a were observed in a minor proportion of our patient cohort, and the median serum levels were lower in comparison to the miR-93, miR-320a and miR-224. This is in line with existing studies, for instance, showing miR-148a as expressed in a low manner in the serum of pre-diabetic individuals [43]. Obesity is frequently associated with PCOS and insulin resistance, and miR-216a has been established as a biomarker for obesity, with lower concentration levels in overweight individuals [22].

Taken together, we detected a robust expression of miR-93, miR-224 and miR-320a as serum-circulating microRNAs, corresponding with the experimental design of a wide range of previous reports on the serum level analyses of these microRNAs as biomarkers in metabolic diseases or tumor diagnosis.

In bivariate correlation analyses, the serum levels of the most microRNAs tested correlated with the serum level of at least one other microRNA. Additionally, the serum levels of miR-224 and miR-216a were significantly correlated with the serum estradiol levels. Recently, estradiol was shown to decrease the amount of serum miR-224 bound to extracellular vesicles in trans-women [44]. An estrogen-regulated lncRNA H19/miR-216a-5p axis has been described in women with endometriosis, and an increased expression of miR-216a enhanced endometrial cell invasion and migration [45]. In addition, we observed that both miR-93 and miR-216a serum levels were correlated with DPP4 levels. Previous reports have shown a regulation of DPP4 by the microRNAs miR-448-3p [46], miR-152 [47] or miR-124-3p [48]; however, no reports on a putative regulatory role of miR-93 or miR-216a on DPP4 exist to date. Our results once again demonstrate the close relationship between deregulated circulating microRNA expression and molecular factors of the menstrual cycle and female fertility.

In our patient cohort, we observed a significant association between a decreased miR-320a serum level and the PCOS phenotype, while no significant associations were found between the individual diagnostic criteria and the serum levels of the tested microRNAs. MiR-320a has previously been shown to be expressed at lower serum levels in PCOS patients [49,50], which supports the association we found in our patients. On the other hand, another study found no association between miR-320 serum levels and the occurrence of PCOS in a cohort of 50 patients in each group [51]. When comprehensively studying the role of miR-320 in PCOS diagnostics in a meta-analysis, it was demonstrated that miR-320 is significantly downregulated in serum, granulosa and cumulus cells; however, this was only based on five studies with small patient numbers [52]. Moreover, in a recent study, a significant lower miR-320 serum level was demonstrated in a cohort of 104 patients with insulin resistance and PCOS compared to healthy controls, and the miR-320 serum levels were inversely correlated with the HOMA index as an indicator of insulin resistance [53]. Besides serum, miR-320a was also studied in the follicular fluid of PCOS patients, wherein miR-320a levels were not significantly different from those in the follicular fluid of Non-PCOS patients [53]. However, lower follicular fluid levels of miR-320a were associated with a smaller number of mature oocytes to be retrieved during ART [54]. On the contrary, Sang and colleagues detected significantly lower levels of miR-320 in the follicular fluid of PCOS patients in a smaller patient group [55]. Taken together, there is growing evidence, confirmed once more in our study on a greater study cohort, that a deregulated miR-320a expression is a molecular feature of the PCOS phenotype. Yet to be explored are the pathophysiological causes and consequences of this deregulated miR-320a expression, which may, for instance, be associated with and actively mediate processes like premature aging and cellular senescence in the vascular as well as in the reproductive compartments in PCOS patients. For instance, such regulatory mechanisms of granulosa cell senescence mediated by microRNAs are already described for, e.g., miR-424-5p [56], miR-200b [57] or miR-125a-3p [58]; however, further research on the interplay of microRNA axes and the pathophysiological features of the PCOS are warranted.

Notably, we also detected a trend towards an association between increased miR-148a serum levels and an androgenic phenotype/hyperandrogenism in our patient cohort. This observation, although not yet supported by other studies in PCOS and female fertility, is in line with the androgen-regulation of the miR-148a expression observed in prostate carcinoma [59,60]. We observed a significant association between the serum levels of all selected IR-associated microRNAs and the occurrence of a pregnancy in subsequent ART cycles. Studies on the predictive potential of serum-bound microRNAs on the establishment of a pregnancy are rare, and the complex process of proper fertilization, embryo development, implantation and fetal growth and development is regulated and influenced by a plethora of different molecular and (patho-)physiological factors. We consider our observation of an association between elevated microRNA serum levels and the subsequent occurrence of a pregnancy not to be of a causal nature but more as a surrogate of the fact that a healthy organism (characterized—besides others—by a well-regulated IR-associated microRNA expression) is more likely to successfully establish a pregnancy.

Analyzing the regulatory effect of hyperglycemia and hyperandrogenism on the expression of miR-93, miR-320a and miR-224 in two granulosa carcinoma cell lines, we detected a significant downregulation of miR-93 and miR-224 after 24 h, which was mainly reversed after 48 h. On the contrary, it was revealed that after 24 h of treatment, miR-320a was significantly upregulated when treated with 100 nM testosterone, while it was downregulated after glucose stimulation. While the activating effect of testosterone on the miR-320a expression remained after 48 h, no expression change upon glucose stimulation was detectable any more. The effects of hyperglycemia on cell viability and proliferation have been extensively studied. Studies have revealed an inhibition of miR-140-3p in endothelial cells [61] or of miR-34a-3p in maturing adipocytes [62] by elevated glucose concentrations. Interestingly, it has recently been shown that human glucagon is tightly regulated by miR-320a expression and that high glucose levels downregulate miR-320a expression in human pancreatic islet cells [63]. Contrary to our results, in prostate cancer, miR-320 was demonstrated to be repressed epigenetically and upon activation to suppress androgen receptor expression by targeting its translation, thereby inhibiting prostate cancer cell growth [64]. In summary, the cell culture experiments revealed a lasting effect of hyperandrogenism, which is a key feature of the PCOS phenotype, on miR-320a expression in granulosa carcinoma cells in vitro.

One limitation of this study is the heterogeneity of the study cohort. Although a relatively large number of participants could be included, stratification according to factors like the BMI was not performed. The statistical analyses showed no association between the serum microRNA levels and overweight or obesity; however, it cannot be ruled out that obesity-associated confounders additionally influence the serum microRNA levels. Another limiting factor may be the number of the analyzed microRNAs, which could be extended to include a broader variety of the IR-associated circulating miRNome. Furthermore, as the data analysis was considered to be exploratory, no statistical corrections were applied. Finally, the results achieved in the cell culture should be further tested and replicated in an animal model of PCOS to further analyze the mechanisms of the PCOS-associated deregulation of serum microRNA expression.

## 5. Conclusions

In conclusion, we report a significant association between lowered serum levels of the IR-associated microRNA miR-320a and the PCOS phenotype and show initial links between the transcriptional regulation of this microRNA and the dysregulated androgen concentrations observed in PCOS patients. These results may be a step towards the sub-characterization of different PCOS metabolic patient types, thereby helping us understand the metabolic changes triggered by PCOS on a molecular level and individually assign personalized treatments to PCOS patients in the clinical reproductive medicine routine.

## Figures and Tables

**Table 1:** Overview of demographic and clinical data of the patient cohort.

	Total Cohort (n = 358 ^1,2^) Median (Range)	Non-PCOS Subgroup (n = 214) Median (Range)	PCOS Subgroup (n = 136) Median (Range)	*p* (Student’s *t*-Test)
Age (years)	32 (19–46)	33 (20–46)	30 (19–40)	<0.001
BMI	23.5 (17.0–47.8)	22.9 (17.0–44.6)	25.3 (17.4–47.8)	<0.001
LH (IU/L)	5.06 (0.23–59.9)	7.94 (0.23–68.5)	11.6 (3.75–59.9)	0.14
FSH (IU/L)	6.07 (1.17–108.0)	6.46 (1.74–108.0)	5.39 (1.17–12.1)	0.03
LH:FSH	1.57 (0.38–5.91)	1.07 (0.38–3.75)	2.20 (0.83–5.91)	0.15
Estradiol (pmol/L)	1221.5 (18.4–7465)	233 (18.4–1776)	194 (73.96–7465)	0.08
Prolactin (mIU/L)	245.0 (107–543)	269 (107–543)	221 (116–396)	0.05
HOMA-IR	1.79 (0.28–18.19)	1.38 (0.28–13.83)	2.47 (0.41–18.19)	<0.001
	**Total** **n (Percentage)**	**Non-PCOS** **n (Percentage**)	**PCOS** **n (Percentage)**	***p* (ChiSquare Test)**
hyperandrogenism	97 (27.7%)	14 (6.5%)	83 (61.0%)	<0.001
dysmenorrhoe	104 (29.7%)	18 (8.4%)	86 (63.2%)	<0.001
polycystic ovaries in ultrasound	78 (22.3%)	5 (2.3%)	73 (53.7%)	<0.001

^1^ No information on PCOS diagnosis for eight patients. ^2^ No information on HOMA-IR for 196 patients.

**Table 2:** Bivariate correlation analyses (Spearman–Rho).

MicroRNA	Correlation with	r_s_	*p*	N
miR-93	DPP4_concS ^1^	−0.281	<0.001	197
	miR-320a	0.519	<0.001	347
	miR-216	0.277	<0.001	285
	miR-224	0.243	<0.001	335
miR-320a	miR-186	0.402	<0.001	192
	miR-93	0.519	<0.001	347
	miR-216a	0.251	<0.001	285
	miR-224	0.425	<0.001	335
miR-216a	Estradiol	0.271	<0.001	186
	DPP4_act ^2^	−0.256	0.002	151
	DPP4_concS ^1^	−0.303	<0.001	147
	miR-148a	0.274	<0.001	220
miR-224	Estradiol	−0.277	<0.001	214

^1^ dipeptidyl peptidase 4 serum concentration. ^2^ dipeptidyl peptidase 4 serum activity.

## Data Availability

The results are available on reasonable request from the last author of this study.
